# Object Classification in Semi Structured Enviroment Using Forward-Looking Sonar

**DOI:** 10.3390/s17102235

**Published:** 2017-09-29

**Authors:** Matheus dos Santos, Pedro Otávio Ribeiro, Pedro Núñez, Paulo Drews-Jr, Silvia Botelho

**Affiliations:** 1 NAUTEC-Intelligent Robotics and Automation Group-Center for Computer Science, Universidade Federal do Rio Grande, Rio Grande 96203-900, Brazil; p.o.ribeiro@furg.br (P.O.R.); paulodrews@furg.br (P.D.-J.); silviacb@furg.br (S.B.); 2 ROBOLAB - Robotics Laboratory, Department of Computer and Communication Technology, Universidad de Extremadura, Cáceres, Extremadura 1003, Spain; pnuntru@unex.es

**Keywords:** underwater sensors, underwater monitoring, underwater surveillance

## Abstract

The submarine exploration using robots has been increasing in recent years. The automation of tasks such as monitoring, inspection, and underwater maintenance requires the understanding of the robot’s environment. The object recognition in the scene is becoming a critical issue for these systems. On this work, an underwater object classification pipeline applied in acoustic images acquired by Forward-Looking Sonar (FLS) are studied. The object segmentation combines thresholding, connected pixels searching and peak of intensity analyzing techniques. The object descriptor extract intensity and geometric features of the detected objects. A comparison between the Support Vector Machine, K-Nearest Neighbors, and Random Trees classifiers are presented. An open-source tool was developed to annotate and classify the objects and evaluate their classification performance. The proposed method efficiently segments and classifies the structures in the scene using a real dataset acquired by an underwater vehicle in a harbor area. Experimental results demonstrate the robustness and accuracy of the method described in this paper.

## 1. Introduction

The ability to construct a map while the robot moves is essential for performing autonomous tasks and has been extensively studied in the literature. Map building allows the robot to develop autonomous skills such as navigation, interaction with environment and self-localization, among others. The scientific community has been studying new ways of representing the map of the environment in the last few decades (one of the most interesting surveys about mapping is found in [1]). Most of the solutions proposed in the literature for this problem are addressed using representations of the spatial structure of the environment (e.g., occupancy cells or geometric features like segment lines). However, it is difficult to perform other tasks successfully using only a spatial representation of the environment. This tendency is now changing, and the scientific community is experiencing an increasing interest in so-called semantic solutions, which integrate semantic knowledge and geometrical information [2].

Recently, several advances in mapping have been achieved. In fact, ground robots that incorporate capabilities for task planning and storing some semantic knowledge in their maps are commonly used (e.g., classification of spaces, such as rooms, corridors or garden, and labels of places and/or objects) [2]. However, very few work has been achieved in underwater robotics where the semantic knowledge of the environment could be applied, for instance, to predict changes and to make high-level decisions. In fact, the mapping problem in underwater robots has been addressed typically by only using geometric information with sonar or Red-Green-Blue (RGB) sensors [3,4,5].

In order to autonomously acquire semantic information from an underwater environment, robots have to be equipped with sensors and a system to extract high-level knowledge from the scene. Typically, RGB sensors have been used in the literature for extracting and characterizing robot’s environment. However, in underwater scenarios, these RGB images provide little information due to water turbidity.

The use of sonar offers the advantage to be invariant to the water turbidity; however, data suffer distortion and noise and thus processing the acoustic signal is still a challenge. The sonar data can be summarized to untextured range data and thus only information about the shape of the detected objects is able to be acquired.

Several works proposed methods to identify objects on acoustic data as [6,7,8,9,10]. However, none of them recognize objects and their semantics in these scenarios. Galceran et al. [6] proposed an underwater object classification on multi-beam sonar data by considering the specific domain knowledge with a limited number of shapes.

In this paper, a study of classification approaches applied to acoustic images is presented with the aim of being used in a localization and mapping system for underwater robots. The proposed study shows how objects can be detected and recognized in the scene allowing a robot to build a map. In addition, it can be integrated with the topological graphs proposed in [11], allowing the construction of more reliable maps for the localization problem, since it would be possible to establish a reliability relation between the objects and their behavior in the environment. For example, static objects such as stones and poles have more confidence than dynamic objects, which change their position over time, such as fish, boats, and swimmers, for the localization problem.

This approach is limited to at least partially structured environments because it is necessary that our approach detects some man-made structures at least in a sparse frequency. Our approach should not be effective in open sea regions where sensory readings are predominantly monotonous.

This paper extends the contributions proposed in [12], with modifications on the segmentation methodology. Now, the local parameter adjustment averages a window of bins in order to find peaks of intensities. These peaks define each local threshold parameter in the segmentation process. In addition, an extended study of the classification algorithms applied to acoustic images is presented, where the Support Vector Machine, Random Trees, and K-Nearest Neighbor classifiers were evaluated.

**Figure 1:**
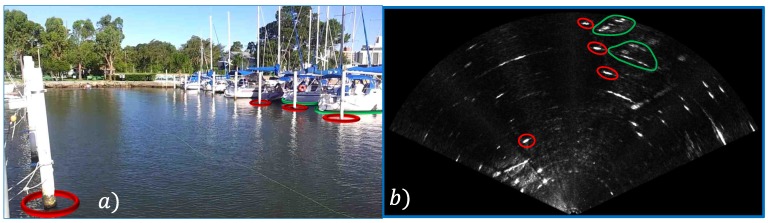
An example of a semantic information that can be extracted from acoustic images collected on a harbor. (**a**) the water surface image with the associated objects; (**b**) the sonar data acquired by an Forward Looking Sonar (FLS) and the segmented areas using colors. We show the same poles in red and the same hulls in green in both images. The acoustic returns that are not highlighted represent structures of the harbor that do not have a vision intersection between the optical and acoustic images. Basically, they also represent poles and boat hulls.

More specifically, this paper describes in detail the experiments and show new results evaluating the solutions on real data acquired by FLS in a harbor. Figure 1 shows an example of the semantic information that can be obtained by the approach. The acoustic images are segmented and their shapes are described geometrically. Then, they are classified into five different classes (Pole, Boat Hull, Stone, Fish and Swimmer) using a tool developed to annotate the sonar data. The annotated data allow the training of supervised classifiers and all created datasets and developed code are made available. The code is available at [13] and the dataset at [14].

## 2. Acoustic Image from a Forward Looking Sonar

The Forward-Looking sonars (FLS) are active devices that produce acoustic waves that propagate through the medium until they collide with an obstacle or are completely absorbed. When a wave collides with an obstacle, a part of its energy is absorbed and a part is reflected. The reflected portion that returns to the sensor is recorded using an array of hydrophones. The round trip of the wave is called *ping*.

**Figure 2:**
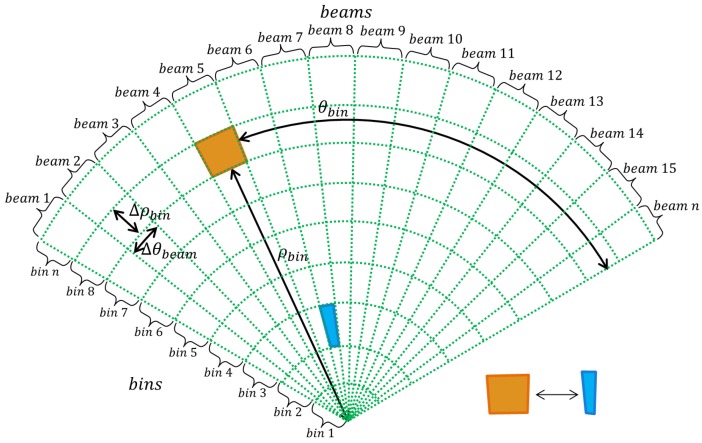
A representative scheme of image formation of an FLS. Each bin can be identified on the polar coordinate system ($\theta_{bin},\rho_{bin}$) and has an angular resolution $\Delta \theta_{beam}$ and a range resolution $\Delta \rho_{bin}$. For this reason, the most distant bins have a lower resolution than the nearest bins. This effect can be visualized on the blue and orange highlight polygons.

The waves captured by the hydrophones are organized according to their return direction and their distance to the reflecting object. Acoustic returns from the same direction belong to the same beam and are called bins. A fan-shaped acoustic image I(X,Y) is one of the ways to represent the beams and bins information recorded between a *ping* interval. Figure 2 shows how an acoustic image is organized with respect to its beams and bins.

Figure 1b shows an example of an acoustic image captured in the harbor of the Yacht Clube of Rio Grande, Brazil. In this image, the pixels are associated with bins, and they are indexed according to their distance $r_{bin}$ and their azimuth direction $\theta_{bin}$ from the sonar, as shown in Figure 2. Due to the FLS conception, the height information of a bin can not be distinguishable and, therefore, the acoustic image is a 2D projection on the horizontal plane of the observed environment.

Although the sonars are almost independent of the water turbidity conditions, they have some characteristics that make it difficult to handle and to extract information, such as:

- The inhomogeneous resolution. The number of pixels to represent a bin varies according to its distance $r_{bin}$ to the sonar. Figure 2 shows two bins overlapped by a box. The orange box covers the farther one and the blue box covers the closer one. The area covered by the orange box is bigger than the blue box. This fact causes image distortion and objects’ flatness.
- The intensity variations of each bin. They are caused by water attenuation, changes in sonar tilt or sensitivity differences between the hydrophone.
- Acoustic reverberation caused when two or more acoustic returns from the same object are captured producing duplicated objects in the image.
- The acoustic shadow effect produced by objects that block the path of the acoustic waves, producing a region without acoustic feedback after the blocking objects. These regions are characterized by a black spot in the image and hide a part of the scene causing occlusion of objects.
- The speckle noise due to the low signal-to-noise ratio caused by mutual interference of the sampled acoustic returns.

Because of these problems, techniques for enhancing, segmenting and describing of acoustic images, specifically developed for FLS, are required.

## 3. Methodology

**Figure 3:**
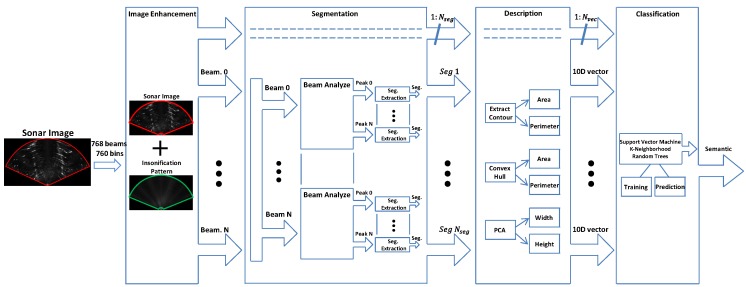
Overview of the proposed semantic system.

The proposed approach for object classification has four steps that include image enhancement, segmentation, description, and classification. A tool has been developed to perform all the steps and to create training data to the supervised classifier. An overview of the proposal is illustrated in Figure 3.

### 3.1. Image Enhancement

We applied in this step an image correction method based on [15]. First, we found the sonar insonification pattern by averaging a large group of acoustic images. After that, the sonar insonification pattern is applied to each image mitigating the effects of the nonuniform insonification and the overlapping problem of acoustic beams.

### 3.2. Image Segmentation

Because of low signal-to-noise ratio and the phenomena described in Section 2, the acoustic images are very noisy and represent a significant challenge faced by our methodology and its quality directly influences the final results.

The main idea of this segmentation approach is to separate the objects of interest from the background. As objects are more efficient than the seabed to reflect acoustic waves, they are characterized by high intensity spots on the images. For this reason, we adopted an approach based on the principles of the sonar operation to detect peaks of intensity.

Each acoustic beam B is analyzed individually, bin by bin.

The average intensity $I_{mean}\left(b,B\right)$ is calculated for each bin *b* of a given beam *B* through Equation (1):

(1) $$I_{mean}\left(b,B\right)=\frac{1}{win_{sz}}\sum_{i=b-win_{sz}}^{b}I\left(i,B\right),$$

where $win_{sz}$ is the window size, in the number of bins, included in the averaging; *b* and *i* are bin identifiers; *B* is a beam identifier; and I(i,B) is the intensity of $i^{th}$-bin of $B^{th}$-beam. The intensity $I_{peak}\left(b,B\right)$ is an offset of $I_{mean}\left(b,B\right),$ as shown in Equation (2).

(2) $$I_{peak}\left(b,B\right)=I_{mean}\left(b,B\right)+h_{peak},$$

where $h_{peak}$ is a constant that determines the minimum height of a peak of intensity. A sequence of bins with an intensity I(b,B) greater than $I_{peak}\left(b,B\right)$ is considered part of a peak and is not considered in the $I_{mean}\left(b,B\right)$ computation. In this sequence, the bin $b_{peak}$ is the bin with the highest intensity. Its intensity $I(b_{peak},B)$ and position $\left(b_{peak},B\right)$ are adopted to adjust the segmentation parameters.

**Figure 4:**
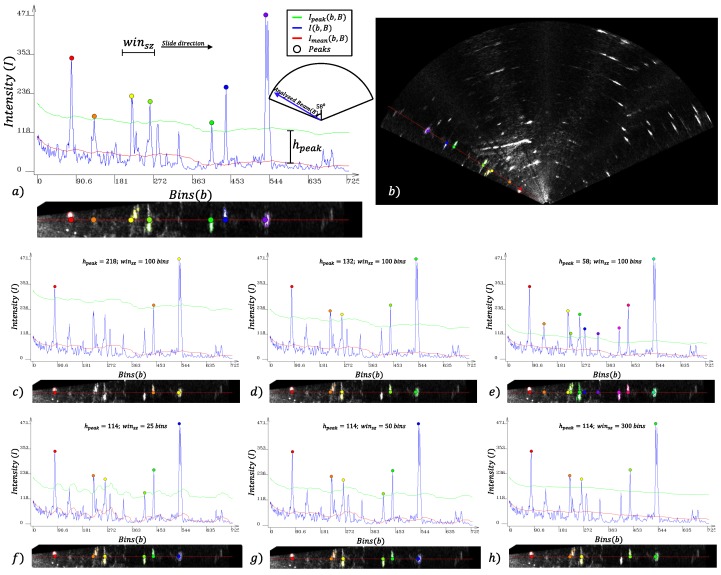
Local tuning parameters for segmentation. The graph represents the analysis of one acoustic beam *B* ($\theta_{bin}=123^{o}$). In this analysis, the peaks of intensity are detected and used to locally adjust the segmentation parameters. The blue line represents the bins’ intensities I(b,B); the red line represents the mean intensities $I_{mean}\left(b,B\right)$, and the green line represents the minimum intensity for peak detection $I_{peak}\left(b,B\right)$. The colored circles represent the detected peaks. As can be seen in (**b**), each segment is extracted based on the intensity and position of the detected peaks in (**a**). The behavior of $I_{peak}$ calculated by Equations (1) and (2) can be observed in (**c**–**e**) when the parameter $h_{peak}$ is changed and in (**f**–**h**) when the parameter $win_{sz}$ is changed.

Figure 4 shows in red the $I_{mean}\left(b,B\right)$ intensities, in blue the I(b,B) intensities and in green the $I_{peak}\left(b,B\right)$ intensities of all bins of a single beam *B*. The peaks detected $b_{peak}$ are represented by colored circles.

From each peak bin $b_{peak}$, a quadruple is defined as {x, y, $I(b_{peak},$ B), $I_{mean}($ $b_{peak},$ B)}, where *x*, *y* is the $b_{peak}$ position in the image. After the detection of all peaks in the image, a search for connected pixels is performed for each peak, initiating on the peak of lower intensity to the highest one. The 8-way connection is adopted as the neighborhood criterion by the breadth-first search algorithm. In this search, all the connected pixels are visited according to the following criterion: A bin $b_{vis}$ is visited if its intensity $I(b_{vis},B)$ is greater than the peak intensity $I_{mean}\left(b_{peak},B\right)$ or its relative distance to a segment border is lower than the parameter $D_{seg}$ in pixels.

**Figure 5:**
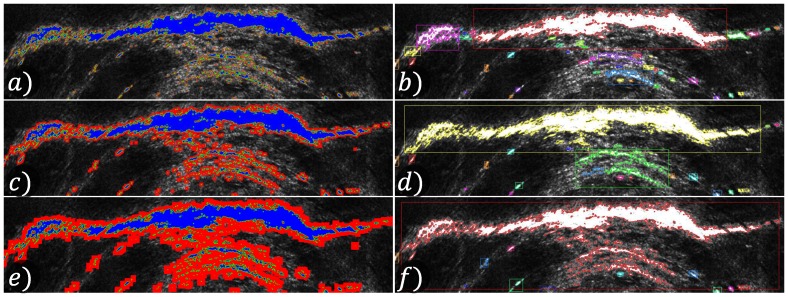
Segment extraction step. After detecting the intensity peaks, a search for connected pixels is performed. These images show the segment extraction of the same image changing the parameter $D_{seg}$. The images on the left show the pixel search process; the visited and those included on the segment pixels are shown in blue, the segment contour pixels are shown in green and the pixels visited on the search to merge nearby segments are shown in red. The right images show the extracted segments. $D_{seg}=1$ was used in (**a,b**); $D_{seg}=4$ was used in (**c,d**) and $D_{seg}=10$ was used in (**e,f**).

The distance criterion is adopted to reduce the multi-segmentation issue of a single object caused when a group of high-intensity pixels is divided by low-intensity pixels. This effect is caused by noise or by acoustic shadows. Figure 5 shows the behavior of the segmentation algorithm by changing the $D_{seg}$ parameter.

### 3.3. Describing Segments

After the segmentation step, each segment is described using a Gaussian probabilistic function and the following information about each segment is computed.

Initially, *width* and *height* are computed using a covariance matrix that relates the *x* and *y* position of each pixel of the segment. The eigenvalues and eigenvectors of the covariance matrix are computed using Singular Value Decomposition (SVD). The width is defined as the largest eigenvalue and height is defined as the second largest eigenvalue.

Furthermore, the segment *area* is computed using Green’s theorem that gives the relationship between a line integral around a simple closed curve. This area is computed using the implementation of the OpenCV library [16]. Finally, we determine the *convex hull area*, the *perimeter*, the *mean intensities* and the *intensities standard deviation* of each segment. Almost all extracted information is geometrical, except the mean and the standard deviation of the intensities.

Based on this information, we defined a ten feature vector 10D features. This is composed of *Inertia Ratio*, i.e., width divided by the height, *mean* and *standard deviation* of the acoustic returns, *segment area* and *convex hull area*. Furthermore, we compute the *convexity*, i.e., the segmented area divided by the convex hull area, the *perimeter* and the *number of pixels* in the segment.

### 3.4. Segment Classification

After the description of the segments, they are classified by a supervised classifier. We evaluated some of the main classifiers: Support Vector Machine, Random Trees and K-Nearest Neighbors adopting the five classes of objects available in our dataset (Pole, Boat Hull, Stone, Fish and Swimmer).

**Figure 6:**
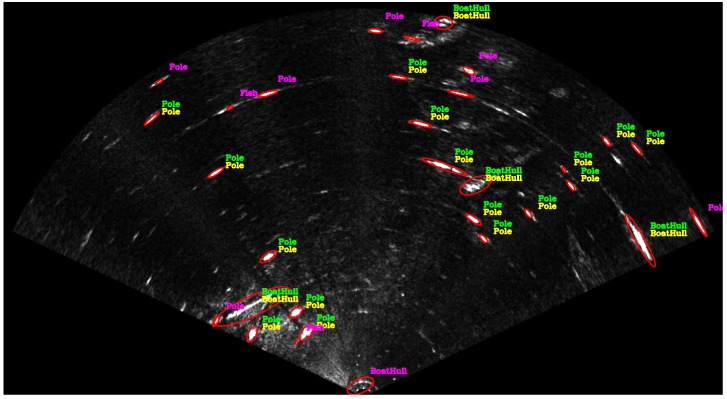
This figure shows how the annotation tool displays the acoustic images. The ellipses in red are automatically detected by the segmentation algorithm, and the yellow labels have been manually defined. After running the classifier training, the labels in magenta, red or green appear to represent the classification assigned by the classifier. The green labels indicate correct classification, red labels indicate incorrect classification, and magenta labels indicate segments without annotation to compare. A video demonstration is available at [17].

The training data is generated by a developed tool that allows the manual annotation of each segment, training the classifiers and saving the manual annotations and the obtained results in text files. Figure 6 shows how the tool displays the acoustic images with the annotated information and the results obtained by the classifier. It is an open source tool developed in C++ using the OpenCV library [16]. The source code is avalible at [13].

#### 3.4.1. Support Vector Machine

The Support Vector Machine (SVM) technique is a classifier that models the data as a k-dimensional vector and defines an optimal hyperplane that best separates the vectors depending on your class. The hyperplane is defined by an optimization algorithm in the training step.

The classification using SVM is based on the libSM library [18]. Its implementation presents several type of kernels that allow us to deal with nonlinear classification. The available kernels are: polynomial, radial basis function (RBF) and sigmoidal kernels. As described in [18], the two kernel function parameters must be defined: γ and *C*. These parameters affect the nonlinearity properties of the kernel functions in the training stage.

These parameters are optimally defined by an auto training function that builds a grid with the classifier performance by varying the two parameters (γ, *C*). The classifier performance is calculated by cross validation, the training data are split into *k* groups, one of them is used for cross-validation and the others train the classifier. A range and discretization step to γ and *C* variation must be defined to build the grid. In this work, the grid is build starting in 0.1 and ending in 60 using a logarithmic step of 0.1 for both parameters γ and *C*.

#### 3.4.2. Random Trees

The Random Trees algorithm introduced by Leo Breiman and Adele Cutler [19] adopt the principle that the combination of learning models increases the classification accuracy. Then, a collection of decorrelated decision trees is adopted to predict the object classification using a vote based approach.

A feature vector is used as the input of each decision tree and its output is a vote. The class that receives the majority of the votes is adopted as the class of the feature vector.

Each decision tree is trained using the same parameters but with different datasets. The training set of each tree is a random selection of the original training set. In addition, one-third of the training set of each tree is left out to get a running unbiased estimate of the classification error and to get estimates of the variable importance of the feature vector.

The OpenCV implementation of Random Trees was used on this work. The main parameters are: *Max. Depth*, defining max depth of the trees, *Min. Sample Count*, defining the minimum samples required to split a leaf node of the tree and *Terminatio Criteria*, defining when to stop the training stage.

#### 3.4.3. K-Nearest Neighbors

The *K*-Nearest Neighbors (KNN) is a non-parametric algorithm that stores all training feature vectors. To predict the class of a new and unknown feature vector, the algorithm finds the *K* nearest feature vectors (neighbors) using an appropriate distance metric to the feature space—in this case, the Euclidean distance. The classification of the unknown feature vector is determined by the majority of the vote of its neighbor’s classes.

When K=1, the algorithm directly assigns the class of the closest neighbor to the unknown feature vector. To avoid cases of a tie, the constant K must not be a multiple of the total number of classes.

## 4. Experimental Results

The experimental results are performed using the acoustic images of an FLS from dataset ARACATI 2014. The training dataset was created using the developed tool. Results are performed using the 10D features as described in Section 3.3.

### 4.1. Dataset ARACATI 2014

The dataset ARACATI 2014 provided by [20] was created using a mini Remote Operated Vehicle (ROV) LBV300-5 manufatured by Seabotix (San Diego, CA, USA) equipped with a Forward Looking Sonar BlueView P900-130 (900kHz) and a Differential Global Positioning System (DGPS). The sonar was configured to cover a range of 30 meters and it was mounted under the robot facing forward with a tilt of 0${}^{\circ }$ degrees.

**Figure 7:**
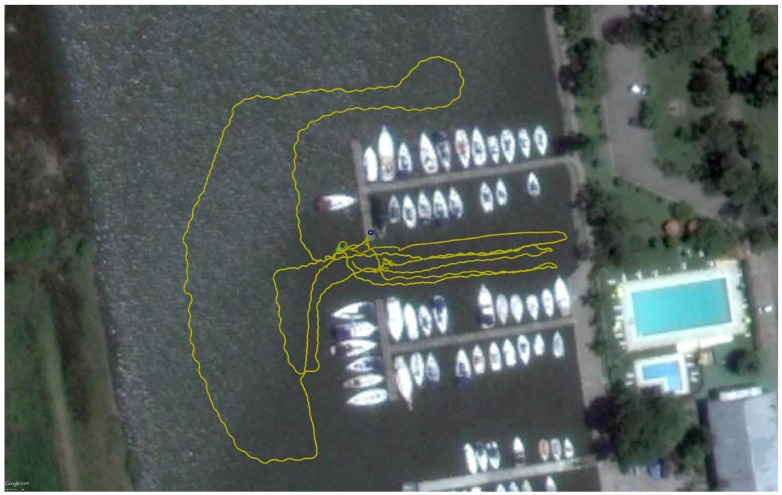
Satellite image of the harbor with the trajectory traveled by the Remote Operated Vehicle (ROV) during the acquisition of the Dataset ARACATI 2014 [20]. Map data: Google, DigitalGlobe 2016.

During the entire path, the ROV remained close to the water surface to not lose the DGPS signal. The sonar stays 40 centimeters from the water surface and four meters from the seabed. The harbor structures such as poles, piers, boat hulls and stones are visible in the acoustic images. Some of them are highlighted in Figure 1. Figure 7 shows a satellite image of the harbor with the trajectory traveled by the ROV.

### 4.2. The Classification Dataset

A new classification dataset was generated on this extended work using the developed tool. The training data consists of a total of 531 labeled segments over 257 acoustic images that were manually classified in one of the five different classes: Pole, Boat Hull, Stone, Fish and Swimmer.

The data were split into two sets: the validation data (20%) and the training data (80%). To avoid the overfitting problem, the validation set is never used in the training stage, and the training set is never used to evaluate the classifiers.

The total number of segments in each class is shown in Table 1. In order to mitigate the effects caused by the unbalanced dataset, our results were generated twice: once with the original unbalanced dataset (the third column) and once compensating the classes in smaller numbers by replicating the segments of the training set (the fifth column).

The parameters adopted in the segmentation algorithm are shown in Table 2, and these parameters were empirically determined by performing several qualitative tests.

The classifier space must be normalized before training to eliminate the range differences between each variable and thus achieve better results. This normalization reduces the scale problem and makes all the dimensions have the same importance to the classifier. The maximum and minimum values adopted in the normalization are shown in Table 3. These values were obtained analyzing the training data.

**Figure 8:**
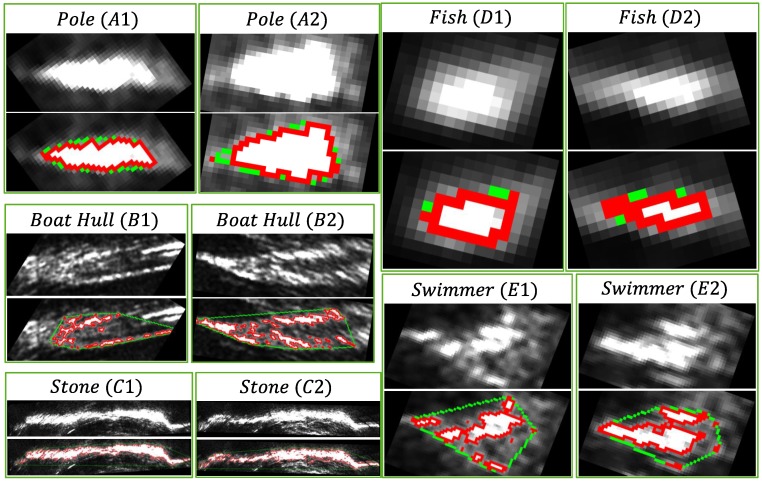
Segmentation results using the parameters of Table 2. Pixels in red represent the segment contour, and pixels in green represent the convex hull. The information extracted from each segment is shown in Table 4. All images are in Cartesian coordinates. This way, we can direct extract geometric information from the segments.

The object recognition in acoustic images is not a trivial task due to the low resolution of the sonar images, as shown in Figure 8. We believe the segment shape and size are the most distinctive features for object recognition, but this information also suffers from image distortion and non-homogeneous resolution problems.

Quantitative information extracted from the segments of Figure 8 is shown in Table 4. The highest and lowest values of each segment are bolded. Some characteristics are easily perceptible, as, for example, the stones are the largest segments, the fish are the smallest segments and the poles are the most convex segments for this dataset.

### 4.3. Best 2D Feature Combination

In this section, we are interested in investigating the best combination of features for the segment classification problem. First, we computed the Pearson correlation coefficient for the 10D features variables unsing the Dataset ARACATI. The result is shown in Table 5.

The Pearson coefficients are shown in percentage. The values close to 100% mean strong correlation, values close to negative 100% mean strong inverse correlation and values close to zero mean no correlation. Each row and column represent one feature variable. The main diagonal always has a value of 100% because it indicates the correlation of the variable with itself. In each line, the symbol (∧) indicates the highest correlation and (∨) indicates the lowest correlation.

It is possible to observe that the variables *area* (6) and *pixel count* (10) have a strong correlation and the size related variables such as *width* (1), *height* (2), and *area* (6) do not correlation with intensity related variables such as the *standard intensity* (4) and *mean intensity* (5). These correlations may be an indication of which values are better for the classification problem, as, for example, *pixel count* and *area* can be considered redundant information for the problem.

We also trained the Random Tree classifier for all combinations of two possible variables (2D Feature) using the replicated training set of Table 1. The obtained hit rate using the validation set can be visualized in Table 6.

The results show that the worst combination is variable *Inertia Ratio* (3), with *Convexity* (8) reaching 51.6% percent of hit rate, and the best is *Mean Intensity* (5), with *Convex Hull Area* (7) reaching 89.83% percent of hit rate at least for the Random Tree classifier.

**Figure 9:**
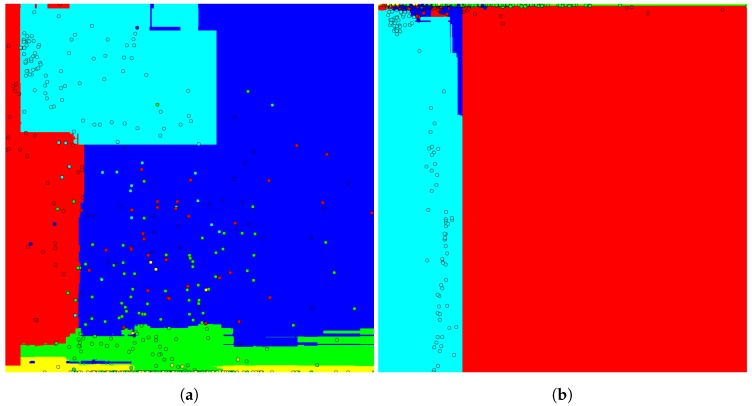
Classification space of the Random Tree Classifier to (**a**) the worst 2D feature combination (*Inertia Ratio* (3) with *Convexity* (8)) and (**b**) the best 2D feature combination (*Mean Intensity* (5) with *Convex Hull Area* (7)).

Because it is a 2D space, an image representing the classification space for the best and the worst feature combination can be generated. The images are shown in Figure 9. The colors represent the objects classes, each circle represents a feature vector and the background represents the classification space.

The classification space is determined by the horizontal and vertical axis of the image that indicates the first and second dimension of the feature vector, respectively. The values increase from left to right and from the top to bottom of the image. In addition, the class colors are represented as, for example, the fish is yellow, the pole is green, the boat hull is red, the swimmer is blue and the stone is cyan.

The presence of feature vector clusters with the same class in the classification space of Figure 9b is notable, making its classification easier, whereas, in Figure 9b, the feature vectors are spread almost randomly, making classification more difficult.

Therefore, the results show that geometric information combined with acoustic intensity information can achieve better results than using purely geometric information.

### 4.4. Results Using 10D Features

In this section, it is investigated which is the classifier that obtains the best results using 10D features. We performed the tests twice: one using unbalanced training data and another using repetitive feature vectors to make the training set balanced as shown in Table 1.

The results using the unbalanced training set are shown in Table 7, Table 8 and Table 9. The results obtained with the balanced training set are shown in Table 10, Table 11 and Table 12.

The results showed that the best performance was achieved by the KNN classifier, with K = 1 reaching a hit rate of 93.57 percent followed by the SVM with the RBF kernel and Random Trees. The balancing technique of the training dataset caused, in general, a decrease in the performance of the classifiers and an improvement in the hit rate of the classes with lower samples in the training set.

## 5. Conclusions

This work presented a complete approach to the object classification problem using the Forward Looking sonar that includes segmentation, description, and classification.

An open source tool for manual annotation and automatic classification of objects in acoustic images has been developed. In addition, some studies were presented based on a real dataset of a harbor area that indicates that the best combinations of features to describe acoustic objects combining geometric and acoustic intensity information. Finally, an evaluation of the Support Vector Machine (SVM), Random Trees (RT), and K-Nearest Neighbor (KNN) classifiers concluded that the KNN classifier with K = 1 is the most suitable object classifier for acoustic images.

Future work will be focused on expanding the study conducted using new and larger datasets with different classifiers, exploring the use of Convolutional Neural Networks (CNNs), integrating the proposed approach with the Simultaneous Localization and Mapping (SLAM) method, and developing an autonomous navigation system using semantic information.

Finally, regarding the presented segmentation method, we intend to evaluate some modifications like replacing the parameter $h_{peak}$ by a standard deviation of intensity and the use of median filter instead of the average of intensity to detect the peak of intensity.

## Figures and Tables

**Table 1:** Dataset information.

Class Name	Total Segments	Training	Repeat	Training wth. Repeat	Total for Validation
Pole	241	192	0	192	49
Boat Hull	63	50	142	192	13
Stone	101	80	112	192	21
Fish	89	71	121	192	18
Swimmer	37	29	163	192	8
Total	531	422	538	960	109

**Table 2:** Segmentation parameters.

Parameter	Value
Bearing	130 degrees
nBeams	768 beams
$H_{min}$	132
$Mean_{sz}$	100 bins
$D_{seg}$	4 pixels
minsegsize	20 pixels
maxsegsize	9000 pixels

**Table 3:** 10D Feature and min, max values on Dataset ARACATI.

Dimension	Feature Name	Min	Max
1	Width	2.49	120.65
2	Height	5.14	641.96
3	Inertia Ratio	0.067	0.892
4	Std. Intensity	61.50	11,446.4
5	Mean Intensity	197.09	2100.16
6	Area	30	45,266
7	Convex Hull Area	30	71,423.5
8	Convexity	0.43388	1
9	Perimeter	17	6169
10	Pixel Count	30	45,266

**Table 4:** Feature values of the segments in Figure 8. In each line, the symbol (∧) indicates the highest value and (∨) indicates the lowest value. The highest and lowest values of each segment are bolded.

Dimension	Pole		Boat		Stone		Fish		Swimmer	
	A1	A2	B1	B2	C1	C2	D1	D2	E1	E2
1	8.3	10.7	31.1	34.2	**78.0∧**	74.7	4.8	**3.1∨**	22.1	14.7
2	26.3	19.7	106.1	130.8	**755.9∧**	772.5	**8.2∨**	9.7	38.3	31.6
3	0.31	0.54	0.29	0.26	0.10	**0.09∨**	**0.58∧**	0.31	0.57	0.46
4	395.2	**647.4∧**	115.9	145.5	157.1	147.3	**73.2∨**	112.4	134.2	136.7
5	291.9	**346.5∧**	**189.9∨**	195.8	201.9	193.3	195.4	204.7	203.0	202.5
6	24	10.5	680.5	1293.5	**31,339.5∧**	28,367.5	**1.5∨**	4	276.5	251.5
7	195	171.5	3457.5	5181.5	**82,339.5∧**	80,409.5	30.5	**26∨**	902.5	555.5
8	0.123	**0.061∨**	0.196	0.249	0.380	0.352	0.049	0.153	0.306	**0.452∧**
9	112.8	47.3	307.5	253.8	3138.1	**25,332.4∧**	**15.2∨**	21.4	115.8	118.2
10	85	66	650	1020	6679	**7192∧**	**24∨**	27	218	166

Feature Dimension Names: 1—Width; 2—Height; 3—Inertia Ratio; 4—Std. Intensity; 5—Mean Intensity; 6—Area; 7—Convex Hull Area; 8—Convexity; 9—Perimeter; 10—Pixel Count.

**Table 5:** Pearson correlation matrix for 10D Feature (%). In each line, the symbol (∧) indicates the highest correlation and (∨) indicates the lowest correlation. The highest and lowest values of each segment are bolded.

Dimension	1	2	3	4	5	6	7	8	9	10
1	100.00	85.02	−30.23	1.97	**−1.71∨**	94.49	93.69	−71.69	**96.70∧**	94.49
2	85.02	100.00	−51.78	2.82	**0.56∨**	93.88	**95.55∧**	−72.13	92.50	93.88
3	−30.23	**−51.78∧**	100.00	**2.65∨**	9.12	−40.02	−40.99	44.79	−40.06	−40.02
4	1.97	2.82	2.65	100.00	**95.03∧**	2.95	2.46	**1.37∨**	2.24	2.95
5	−1.71	0.56	9.12	**95.03∧**	100.00	1.00	0.92	12.98	**−0.29∨**	1.00
6	94.49	93.88	−40.02	2.95	**1.00∨**	100.00	99.53	−62.44	98.96	**100.00∧**
7	93.69	95.55	−40.99	2.46	**0.92∨**	**99.53∧**	100.00	−63.76	99.06	99.53
8	−71.69	**−72.13∧**	44.79	**1.37∨**	12.98	−62.44	−63.76	100.00	−66.41	−62.44
9	96.70	92.50	−40.06	2.24	**−0.29∨**	98.96	**99.06∧**	−66.41	100.00	98.96
10	94.49	93.88	−40.02	2.95	**1.00∨**	**100.00∧**	99.53	−62.44	98.96	100.00

Feature Dimension Names. 1—Width; 2—Height; 3—Inertia Ratio; 4—Std. Intensity; 5—Mean Intensity; 6—Area; 7—Convex Hull Area; 8—Convexity; 9—Perimeter; 10—Pixel Count.

**Table 6:** Performance of 2D feature combination (%).

Dimension	1	2	3	4	5	6	7	8	9	10
1	X	87.57	80.22	83.23	83.23	86.81	87.94	80.22	86.44	86.81
2	87.57	X	80.60	87.38	87.57	85.12	86.81	79.28	81.73	85.12
3	80.22	80.60	X	67.60	66.10	84.74	88.79	51.60	81.35	84.74
4	83.23	87.38	67.60	X	61.01	87.57	89.07	82.29	87.38	87.57
5	83.23	87.57	66.10	61.01	X	87.38	89.83	78.90	88.70	87.38
6	86.81	85.12	84.74	87.57	87.38	X	85.31	83.99	84.93	83.05
7	87.94	86.81	88.79	89.07	89.83	85.31	X	84.55	84.18	84.93
8	80.22	79.28	51.60	82.29	78.90	83.99	84.55	X	81.73	83.99
9	86.44	81.73	81.35	87.38	88.70	84.93	84.18	81.73	X	84.93
10	86.81	85.12	84.74	87.57	87.38	83.05	84.93	83.99	84.93	X

Feature Dimension Names. 1—Width; 2—Height; 3—Inertia Ratio; 4—Std. Intensity; 5—Mean Intensity; 6—Area; 7—Convex Hull Area; 8—Convexity; 9—Perimeter; 10—Pixel Count.

**Table 7:** Unbalanced 10D Feature results—Support Vector Machine.

Parameters					Result Hit (%)					
Kernel	γ	*C*	Degree	Coef0	Total	Pole	Boat	Stone	Fish	Swimmer
Linear	-	-	-	-	84.40	81.63	84.61	100	88.88	50
Polynomial	41.55	28.45	0.49	274.4	77.98	79.59	53.84	100	88.89	25
RBF *	41.55	28.45	-	-	77.98	79.59	30.76	100	100	37.5

* RBF—Radial Basis Function.

**Table 8:** Unbalanced 10D Feature results—Random Trees.

Parameters		Result Hit (%)					
Max Depth	Min Sample Count	Total	Pole	Boat	Stone	Fish	Swimmer
5	10	83.48	83.67	69.23	100	100	25

**Table 9:** Unbalanced 10D Feature results—K-Nearest Neighbors.

Parameters	Result Hit (%)					
k	Total	Pole	Boat	Stone	Fish	Swimmer
1	81.65	85.71	30.76	100	94.44	62.5
8	53.21	48.97	46.15	100	38.88	0
28	51.37	53.06	46.15	100	16.67	0

**Table 10:** Balanced 10D Feature results—Support Vector Machine.

Parameters					Result Hit (%)					
Kernel	γ	*C*	Degree	Coef0	Total	Pole	Boat	Stone	Fish	Swimmer
Linear	-	-	-	-	75.22	67.34	38.46	100	94.44	75
Polynomial	41.55	28.45	0.49	274.4	66.97	51.02	84.61	100	88.89	0
RBF	41.55	28.45	-	-	89.90	83.67	84.61	100	100	87.5

**Table 11:** Balanced 10D Feature results—Random Trees.

Parameters		Result Hit (%)					
Max Depth	Min Sample Count	Total	Pole	Boat	Stone	Fish	Swimmer
5	10	78.89	67.34	61.53	90.47	100	100

**Table 12:** Balanced 10D Feature results—K-Nearest Neighbors.

Parameters	Result Hit (%)					
k	Total	Pole	Boat	Stone	Fish	Swimmer
1	93.57	85.71	100	100	100	100
8	61.46	44.89	61.53	95.23	66.67	62.5
28	57.25	49.37	23.80	91.08	83.14	10.81
